# From equity to solidarity: how care and relationality transform the debate on justice in genomics

**DOI:** 10.1007/s12687-026-00932-5

**Published:** 2026-08-11

**Authors:** Barbara Prainsack

**Affiliations:** Department of Political Science, University of Vienna, Universitätsstraße 7, Wien / Vienna, 1010 Austria

**Keywords:** Solidarity, Equity, Care, Relationality

## Abstract

Equity has become central to genomics, yet dominant approaches - focused on fair representation and distribution - often overlook the relational and structural conditions through which genomic practices are produced and experienced. This paper reframes genomic justice by adding care and solidarity to the conversation. Care highlights lived experience, interdependence, and the relational work required to make genomic knowledge meaningful, while solidarity can help to create institutional arrangements grounded in shared vulnerability, indirect reciprocity, and non-humiliation. The paper applies this framework across key domains, including participation, data governance, benefit-sharing, and global collaboration, proposing shifts toward community-led governance, relational data stewardship, and long-term collective returns. It outlines practical design principles that embed equal dignity, trustworthiness, and mutual accountability into genomic institutions. Community genetics is particularly well positioned to operationalise this approach, given its grounding in families and communities and its emphasis on ongoing relationships. Strengthening these practices can support more just, caring, and resilient genomic futures.

## Introduction

Equity has become a central concern in genomics (understood, here, as including both genomic research and medicine). Calls for equitable access to genomic technologies, diversification of genomic datasets, and fairer distribution of research benefits are now standard features of policy briefs, funding calls, and research-ethics discourse. Yet despite decades of equity-oriented initiatives, the field continues to reproduce and even amplify structural inequities (Atutornu et al. 2022; Dolan et al. 2024; Rebbeck et al. 2022). Disadvantaged communities have often been poorly served by their societies in healthcare and other ways and excluded from biomedical research; they remain underrepresented in genomic datasets (e.g. Corpas et al. 2025; Sirugo et al. 2019). Benefits disproportionately accrue to commercial actors or well-resourced health systems (e.g. Mc Cartney et al. 2024). Globally, genomic data still flow largely from the Global South to the Global North, while clinical and economic benefits rarely travel in the opposite direction (Ewuoso et al. 2022; Madden et al. 2024). This raises a fundamental question: Are the categories and instruments used to address inequities in genomics sufficient and suitable to address the problems at hand?

This paper argues that they are not. More specifically, the dominant frameworks for equity in genomics rely – directly or indirectly - on distributive logics, concerned with the fair allocation of risks and benefits as well as fair representation and access. These approaches, while important, are insufficient for grappling with the relational and structural practices and institutions that shape benefits and harms in genomic medicine.

The paper starts by highlighting the role of care as an ethical, analytical and catalytic lens that foregrounds human needs, context, and interdependence, complementing the more abstract concern with the fair and consistent application of principles and rules that has traditionally characterised justice and equity-based frameworks. At the same time, the very features that make care ethically compelling - its attentiveness to specific relationships, contexts, and needs - also make it difficult to scale beyond specific settings. The norms that sustain caring relationships can often not be extended to larger and more anonymous social, political, or institutional contexts. The paper therefore introduces solidarity as a complementary principle that preserves care’s responsiveness to human vulnerability while translating it into durable institutional arrangements capable of coordinating action across larger populations. It then applies this combined solidarity-based framework to key domains of genomic practice - including participation, data governance, benefit-sharing, and global collaboration - arguing that it provides a stronger foundation for building genomic infrastructures that are more equitable in a deeper and wider sense of the word.

### A note on equity and justice in genomics

In this paper, I treat equity and justice are closely related but distinct concepts. I understand equity to refer to an outcome or principle of fair access, whereas justice is a broader framework concerned with the social, political, and institutional conditions that produce (or undermine) fairness – and the rules that need to be followed to achieve the latter.

In genomics, the focus of debates on equity is on reducing disparities and ensuring that people with different needs have fair opportunities to benefit from genomics (e.g. Madden et al. 2024). While these debates shade into discussions on justice, the point of gravity within the latter is different insofar as justice is more concerned with the *rules* for fair distribution and their reference points. One of the concerns of this paper is to broaden the notion of equity to encompass not only concerns of justice but also concerns related to care and solidarity.

## Where dominant frameworks for genomic equity fall short

The justice-based discourse on genomics, which gained prominence in the 1990s alongside the Human Genome Project and subsequent global health equity debates, has broadened the field’s understanding of what responsible genomic science requires (e.g.; Benjamin 2009; Bliss 2012; Fullwiley 2007, 2015; Kraft et al. 2018; Lee et al. 2008; Nelson 2016; Reardon 2009, 2019; Siermann et al. 2025). It has also reshaped practices on the ground: It has helped researchers, funders, and policymakers to confront inequities in participation, data representation, and benefit sharing, and to acknowledge that genomic knowledge is produced and acted upon within social and political structures that do not affect all communities equally (e.g. Hardcastle et al. 2024). It has influenced funding priorities and encouraged long-term capacity building in low-resource settings rather than extractive, short-term studies (e.g. Bentley et al. 2017; Munung et al. 2017).

But the dominant justice-focused discourse has limits. Much of the genomics equity literature approaches justice through a distributive lens: Who is represented in genomic datasets? Who gains access to genomic screening or personalised therapies? Who receives benefits from genomic research? How do we ensure fair inclusion? Although more recent scholarship on justice has expanded the distributive paradigm to encompass goods such as recognition, participation, power, and social standing (e.g. Fricker 2007; Hanlon 2024; Young 1990; Zimm et al. 2024), they remain, I argue, primarily concerned with the allocation of socially valuable goods and opportunities. Many supposedly non-distributive theories ultimately concern the allocation of socially valuable things, such as recognition in recognitional or epistemic justice, decision-making power in the case of procedural justice, or trust, responsibility, and social membership in the case of restorative justice. In other words, the central question is still who gets what, even if the ‘what’ extends beyond material resources.

This focus leaves less room for questions that are not distributive, but that concern notions of care and solidarity. In genomics, participation and benefit are shaped not only by access to resources but also by relationships of trust, shared responsibility, institutional stewardship, and histories of exclusion (e.g. TallBear 2013; Tsosie et al. 2021a, b; see also Carroll et al. 2020). Even autonomy is not merely an individual capacity that can be protected through consent, but something that is enabled - or constrained - by social environments and institutional arrangements (Baylis et al. 2008; Dove et al. 2017; Mackenzie and Stoljar 2000). When these relational factors are ignored, even well-intentioned equity initiatives may reproduce the very hierarchies they aim to dismantle.

Moreover, the language of justice and representation has been coopted for other purposes.^1^ Calls to diversify genomic datasets through the inclusion of populations from underrepresented regions are sometimes motivated by epistemic and clinical interests rather than by commitments to global justice (e.g. Carroll et al. 2022; Tsosie et al. 2021b). Expanding representation can improve the classification of genetic variants, for example by helping to determine whether variants that appear rare in European-derived datasets are in fact benign in other populations. While these are important scientific advances, framing such efforts primarily in terms of justice risks obscuring the extent to which they are also driven by the needs and priorities of well-resourced research systems.

Another reason for distributive justice-based approaches to equity running into their limits this is that often assume individuals to be the primary units of moral agency (e.g. Yang 2025). This view reflects a deeply individualised ontology characteristic of neoliberal governance (and, as some authors would argue, of Western thinking more broadly, see e.g. Battersby 2007; Douzinas 2000; Epstein 2013; Richardson 2007; Taylor 1985), where social arrangements are assessed by their impact on (presumably) bounded individuals rather than relationships, communities, or structures. The result is a policy discourse that treats equity as a matter of ‘getting the distribution right’, while leaving untouched the systems that produce inequities in the first place (Tuhiwai Smith 2012; Wilson et al. 2021; see also Epstein 1996). Genomics’ entanglement with commercial interests, its deep reliance on data extraction, and the political–economic power relations that shape how genomic knowledge is produced, governed, and valorised often remain outside distributive debates.

Next to other research fields such as care ethics, Indigenous data solidarity, or feminist scholarship, community genetics has long offered an alternative to individualised and distributive framings by emphasising that genetic information is never solely about isolated persons but it is embedded in families, kinship networks, and broader social relations (e.g. ten Kate 2005; Raz 2010). Emerging at the intersection of clinical genetics and public health, and shaped in part by critical reflection on the legacy of eugenics, community genetics has developed as a field concerned not only with the delivery of genetic services to populations, but with the social organisation, governance, and ethical orientation of those services. Rather than treating genetic risk, decision-making, or benefit as matters of individual choice or entitlement, it foregrounds shared interests, interdependence, and the infrastructures - familial, institutional, and societal - through which genomic information is produced, interpreted, and acted upon.

This perspective moves beyond treating ‘the community’ as a target of intervention and instead understands communities as sites where meanings of risk, responsibility, and health are collectively produced and experienced. It highlights how participation in genomics is shaped by relationships of trust, care, and dependency, and how these relationships are sustained - or undermined - by health systems and broader institutional arrangements. Community genetics has also consistently emphasised that ethically robust genomic practice depends not only on access to tests or resources, but on the cultivation of trust, reciprocity, and long-term engagement between communities and institutions. Participation in genomics, on this view, is not a discrete, individual decision but part of ongoing, interdependent processes involving family members, care networks, and collective forms of deliberation and support.

In this sense, alongside care ethics and feminist as well as pertinent Indigenous scholarship, community genetics can be understood as an articulation of concerns that prefigure more recent critiques of institutionalised carelessness and the uneven distribution of responsibility in complex societies (see next section). In its ideal form, community genetics - by recognising communities not merely as sites of recruitment or data extraction but as actors with their own epistemic authority, moral claims, and situated forms of expertise (e.g. Schmidtke and Cornel 2020) - draws attention to the conditions under which relationships of care are enabled or eroded. This includes acknowledging asymmetries in power and resources, as well as the ways in which institutional practices can fragment responsibility, displace burdens, or fail to sustain the social relations on which genomic medicine depends.

Bringing this relational and care-sensitive perspective more fully into mainstream genomics would align ethical governance with the realities of how genomic information circulates and acquires meaning in everyday life. It would also help shift the field away from extractive and instrumental models toward approaches grounded in mutual responsibility and the active maintenance of relationships of care - thereby addressing not only questions of distribution, but the deeper institutional and cultural conditions that shape how responsibility for genomic knowledge and its consequences is organised.

## Care as a foundation for rethinking genomic equity

As noted, recent scholarship argues that many contemporary social problems arise not merely from a lack of resources or poor intentions but from deep-seated institutional and cultural failures to recognise and sustain the forms of care that societies depend on (e.g. Chatzidakis et al. 2020a, b; Dowling 2022; Fraser 2022; Graziano et al. 2025; Peterie and Broom 2024; Peterie et al. 2026). This work builds on Indigenous and care ethics scholarship that treats care not as a peripheral activity but a fundamental democratic practice: societies thrive when they attend to people’s needs, vulnerabilities, and interdependencies, and they falter when these are ignored (e.g. Boulton and Brannelly 2015; Whyte and Cuomo 2016; Yates 2021). Tronto’s concept of ‘privileged irresponsibility’ (Tronto 2013), for example, describes how those with power can distance themselves from hardships that others suffer, as well as from the consequences of their actions, leaving the burdens of care to those with fewer resources or to no one at all. Insofar as such privileged irresponsibility becomes institutionalised and shapes political and economic institutions, societies suffer from *structural carelessness* (see also Kotiswaran 2021) - a systemic failure to organise social, political, and economic life around the realities of human and ecological dependency. Rather than isolated moral lapses, such carelessness reflects institutional arrangements that devalue care work, fragment responsibility, and prioritise efficiency, innovation, or profit over collective wellbeing.

Virtually all societal crises, these authors argue - such as loneliness, environmental degradation, democratic decline, as well as the inequities reproduced in health and technology systems - can be traced back to this structural erosion of care. Even in states that had strong welfare institutions a few decades ago, governments have hollowed out many of these institutions and eroded the capacity of people to care for themselves and for others. As a solution, authors such as *The Care Collective*, for example, propose that care must become a central organising principle of political and economic life (Chatzidakis et al. 2020a). Literature across sociology, feminist theory, and political philosophy resonates with this shift: structural carelessness is increasingly understood as a root cause of social fragmentation, inequity, and democratic malaise, while a politics of care is positioned as essential for building more just, sustainable, and resilient societies (e.g. Brown 2015; Escobar 2018; Fraser 2022; Kittay 2019).

How does this relate to genomics? Many genomic practices and institutions are situated within political economies that devalue relationality, attentiveness, and human flourishing. In genomics, structural carelessness, where present, manifests itself in the form of burdens placed on participants to consent, contribute data, and manage risks; in the lack of long-term investment in community partnerships and trust-building; in data extraction logics that treat genomic information as a commodity rather than a relational asset; and on a strong reliance on individual responsibility in interpreting and acting on genomic risk. Rather than merely fair distribution, addressing these problems requires a shift in how institutions understand and organise relationships. And here, care has a pronounced strength – and also a corresponding weakness.

I will start with the strengths. Insofar as care refers to practices and arrangements that sustain people’s well-being through attentiveness, responsiveness, and relational responsibility, it can offer an alternative moral grammar for genomics. A care perspective emphasises the embodied and lived experiences of participants and patients. It situates genomic choices within networks of social relations, dependencies, and vulnerabilities and challenges individualised notions of risk and responsibility. It foregrounds context, including structural and historical forces, and values forms of everyday relational labour that tend to remain invisible within technical and regulatory frameworks. Next to aforementioned work within Indigenous and feminist scholarship, this emphasis resonates strongly also with the sociological tradition developed by Anselm Strauss and colleagues, who conceptualised *patient work* as the wide range of tasks that patients and their families perform to manage illness, navigate healthcare systems, and integrate medical demands into everyday life (Strauss et al. 1982, 1997). Strauss and colleagues’ work showed that care is not confined to clinical encounters, but extends into homes, workplaces, and social relationships, where patients engage in often unrecognised labour to make medical interventions workable. As I argued in another place (Prainsack 2017), patient work intensifies, rather than recedes, in the context of genetics and genomics. Genomic medicine frequently shifts responsibility for interpretation, decision-making, and long-term management onto patients and their families who are expected to understand complex probabilistic information, assess future risks, communicate genetic findings to relatives, and make ongoing decisions about surveillance and prevention. What is framed institutionally as empowerment or personalisation thus often entails significant cognitive, emotional, and relational labour (e.g. Aramoana et al. 2020; Arteaga Pérez 2022; Hudson et al. 2020). This – mostly unpaid, and often invisible labour - is constitutive of how genomic medicine functions in practice. Attending to ‘patient work’ – in this very broad sense of the word - therefore draws attention to how genomic knowledge is enacted through relationships, how burdens are distributed, and how responsibilities are moralised and privatised. By making patient work visible, a care-based approach challenges accounts of genomics that focus narrowly on information, consent, or choice, and instead highlights the relational infrastructures and sustained labour required to live with genomic knowledge over time.

More broadly, what a care perspective brings to genomics is a shift of attention toward the emotional and relational labour involved in consenting to data use, the quality of communication about risk and uncertainty, the relational consequences of genomic knowledge within families and communities, the supports people need to use genomic information for their well-being, and the responsibilities of institutions to provide stable, patterned, and long-term support.

At the same time, care has limits when it is supposed to scale up to large-scale institutions (Prainsack 2026). One of the strengths of care is that it attends to specific needs of specific people in specific contexts. When it comes to institutionalisation, this strength becomes a limit: care does not readily scale without losing its context sensitivity or being transformed into standardised judgements about what others need. Put differently, because care often arises in dyadic relationships (care-giver/care-receiver), care relationships are asymmetrical: They emerge because one party has needs that another party recognises, takes responsibility for, and responds to. Asymmetries in care relationships are not problematic per se; they can become fraught, when these relationships are scaled up to institutions serving broader groups of people. Institutions cannot replicate these dyadic dynamics without becoming paternalistic or flattening relational nuance. In genomics, while care plays a crucial role in clinical encounters, counselling practices, and ongoing support of patients and families, scaling up care into genomic institutions bears risks – such as paternalistic interpretations of what communities need, or one-way flows of care from institutions to ‘beneficiaries’. For these reasons, care alone is not a sufficient complement to justice in the creation of genomic systems capable of sustaining equity. This is where solidarity becomes essential. 

## Why solidarity is indispensable for genomic equity

As we have argued elsewhere (Prainsack and Buyx 2011), solidarity is best defined as relational practice that is based upon what people consider themselves as having in common with others. Unlike care, which expresses itself in asymmetrical relationships and practices (which is one of the reasons for its strength in *attending to specific needs*), solidarity emerges where people recognise commonalities with others amidst difference. Solidarity thus provides a conceptual foundation for designing genomic systems that do not only aim to distribute access and benefits in a fair manner (which is the focus of justice) or respond not only to individuals’ needs (which is the strength of care) but to shared vulnerabilities and collective wellbeing.

Crucially, this recognition of commonalities – or more precisely of ‘similarities in a relevant respect’ (Prainsack and Buyx 2011) within solidarity-based frameworks does not presuppose ‘sameness’ of identity, values, or life situation. Solidarity does not require people to be alike in terms of all or most identity markers, to agree with one another, or to share a comprehensive worldview. Rather, it rests on the acknowledgement that, in specific contexts, amidst all the differences, there are important similarities: patients needing care, workers being exposed to economic precarity, citizens affected by climate risks, or people whose data are being extracted. Policies and institutions often have good reasons to foreground differences - for example, when allocating healthcare according to clinical need, designing disability accommodations, or addressing the particular disadvantages experienced by historically marginalised groups. Yet there are also contexts in which institutions must foreground commonalities rather than differences. This is especially important where shared vulnerabilities, mutual dependencies, or collective goods are at stake. In genomics, this may appear counterintuitive because the field is often framed in terms of biological difference, individual risk profiles, and personalised interventions. However, the infrastructures on which genomics depends - from biobanks and data repositories to standards, public trust, and long-term stewardship - are shared, collective endeavours. They rely on what people have in common: shared vulnerabilities, shared interests in trustworthy institutions, and a shared dependence on knowledge infrastructures that can only be sustained collectively. A solidarity-based perspective therefore complements the emphasis on biological and individual differences by drawing attention to the common institutional conditions that make genomic knowledge possible and beneficial for all.

Solidarity can take shape at different levels of social life: in interpersonal practices between individuals, in shared norms within groups and communities, and in the policies and institutions that organise collective life (Prainsack and Buyx 2011, 2017). It is at this institutional level that solidarity acquires particular significance for genomics. Institutions such as universal healthcare exemplify solidarity because they are built on the recognition of a relevant commonality: despite all differences, everyone is vulnerable to illness and may require care at some point in their lives. Likewise, welfare institutions rest on the premise that all members of society are entitled to the conditions necessary for a dignified life, even if their needs, capacities, and life trajectories differ considerably. Rather than treating solidarity as a sentiment or moral aspiration, this understanding conceives of it as something that can be designed into institutions.

Institutions of solidarity should embody three core design principles (Prainsack 2026): they should recognise the equal dignity of all persons and their capacity to contribute; they should be organised around indirect reciprocity, acknowledging that people contribute in different ways and at different times; and they should minimise humiliation by ensuring that receiving support does not undermine a person’s standing or self-respect (Margalit 1996, 1997). These principles provide a framework for rethinking the governance of genomics in ways that complement care while enabling it to be sustained at scale. I will now discuss what each of these principles mean for genomics.

### Design principle 1: respect for equal dignity and “everybody’s” capacity to contribute

Following the design principles of institutions of solidarity, genomic systems grounded in solidarity must begin from the premise that all people possess equal dignity and a capacity to contribute to society, even when such contributions differ in form, scale, or timing. This requires moving beyond deficit-oriented framings that depict certain populations as ‘hard to reach’, disengaged, or lacking in relevant resources. Instead, solidarity-oriented genomics recognises community knowledge, lived experience, and relational expertise as valuable forms of contribution alongside scientific and technical inputs. Institutional infrastructures should therefore be designed in ways that do not systematically privilege already well-resourced actors, whether academic, clinical, or commercial, and that enable meaningful participation across diverse social positions. For example, this implies designing recruitment, consent, and governance processes that are co-developed with communities, remunerate participatory labour, and accommodate different forms of engagement rather than presuming a single, standardised model of contribution. It also entails treating newcomers - such as migrants or recently incorporated communities - as full members of genomic publics, rather than as peripheral or conditional participants whose inclusion is seen as optional or secondary.

This, in turn, has implications on how access to basic healthcare systems and to advanced genomic medicine are organised, both formally and practically. Solidarity-based genomic systems cannot be meaningfully realised in the absence of equitable access to high-quality healthcare. Achieving equitable benefits from genomic advances depends critically on the context of broader healthcare infrastructures and systemic equity issues: genomic medicine’s promises are mediated by access to high-quality health services and social determinants of health rather than technological sophistication alone (Farmer et al. 2013; Green et al. 2023, 2024; Halbert 2022; Khoury et al. 2018, 2022; Vogt and Hofmann 2022). Where access to basic healthcare is uneven, genomics risks amplifying existing inequalities by primarily benefiting those who are already well served by the health system. Ensuring universal access to comprehensive, high-quality healthcare is therefore not merely a complementary goal but a foundational condition for the just integration of genomics. Without such access, appeals to participation, contribution, or shared benefit remain largely symbolic, as large segments of the population are structurally excluded from translating genomic knowledge into improved health outcomes.

### Design principle 2: indirect reciprocity and generalised trust

In solidarity-based institutions, trust does not emerge from expectations of immediate or symmetrical exchange, but from experiences of reliable institutional support over time. Applied to genomics, this implies a commitment to transparency about how data are used, by whom, and for what purposes, as well as long-term investment in relationships with participating communities. Trust is undermined when access to genomic services or benefits is made conditional, punitive, or contingent on narrowly defined forms of compliance. Instead, institutions must foster feedback mechanisms that reflect communities’ priorities and concerns, demonstrating responsiveness rather than one-off consultation. In this sense, trust is not something to be secured once, but something that must be continuously earned through consistent institutional practice.

### Design principle 3: non-humiliation

A central requirement of institutions of solidarity is the avoidance of humiliation - that is, institutional practices that deny people’s dignity or position them as inferior. In genomic contexts, humiliation may arise from paternalistic modes of communication that disregard community agency, or the dismissal of personal or local needs and values. It may also occur when groups are excluded due to perceived non-compliance with institutional norms, or when genomic systems disproportionately benefit populations that are already privileged. Solidarity therefore demands proactive institutional design aimed at preventing humiliation by embedding respect, agency, and mutual recognition into governance structures, data practices, and modes of engagement. Only by doing so can genomic institutions sustain justice and legitimacy across diverse social contexts.

## Toward a solidarity-and-care framework for genomic equity: examples

A paradigmatic example of genomics embedded in solidaristic infrastructure is England’s NHS Genomic Medicine Service. England has invested heavily in advanced genomic technologies, including whole-genome sequencing and integrated national datasets. These technologies are institutionalised within a universal healthcare system that guarantees access based on clinical need rather than ability to pay (although this principle has been hollowed out by many years of underfunding, staff shortages, and partial privatisation; see e.g. Gibbons 2025; Palmer 2025). Genomic testing for rare diseases and cancer is accompanied by publicly funded counselling, follow-up diagnostics, and treatment pathways. This enables genomic knowledge to translate into tangible health benefits across socio-economic groups.

From a solidarity perspective, the NHS context is instructive. The system pools risks, costs, and benefits across the population and over time, operating on principles of indirect reciprocity: people contribute via the (level of) tax that they pay, and they draw services based on need. Genomic medicine inherits this logic. While not all participants benefit immediately or directly, the institutional framework is designed to ensure that benefits circulate within the collective. For a long time, the NHS also provided a stable institutional structure that embodied and commanded trust, accountability, and continuity - conditions that made participation meaningful rather than episodic or extractive. The combination of respect for people’s dignity, indirect reciprocity, and non-humiliation, which the NHS embodied, are arguably also one of the reasons for the NHS having been widely seen as ‘the closest thing we have to a religion’ (Spence 2017).

A contrasting picture emerges in the United States, despite the availability of world-leading genomic technologies and ambitious precision-medicine initiatives such as the All of Us Research Program (All of Us Research Program Investigators 2019). The US has immense sequencing capacity and research expertise, yet healthcare access remains fragmented, insurance-dependent, and unevenly distributed. Even when genomic testing is offered through research participation, downstream services - specialist consultations, preventive interventions, targeted therapies - may be inaccessible or prohibitively expensive for many.

In this context, genomic information often benefits those already well served by the healthcare system, while others receive risk information without the means to act on it. Responsibility is shifted onto individuals, who are expected to manage genetic risk, communicate results to relatives, and navigate complex care pathways in the absence of reliable institutional support. This not only reinforces existing inequalities but also substantially increases the burden of patient work. Genomics already relies on considerable labour by patients and their friends and families - understanding test results, coordinating care across specialties, sharing information with relatives, and making difficult decisions under conditions of uncertainty. Where institutional support is weak, this work expands further to include securing access to testing, navigating fragmented healthcare systems, negotiating insurance coverage or out-of-pocket payments, and identifying appropriate follow-up care. In this manner, genomics risks amplifying existing inequalities, turning health advantages into cumulative ones.

This comparison shows that without solidaristic healthcare infrastructures, genomics is unlikely to be equitable, no matter how much emphasis is put on fair representation and distribution. The reason for this is that neither representation nor distribution can be meaningfully specified in the abstract, or independently of the institutional contexts in which people’s needs, vulnerabilities, and claims are articulated. It is often unclear what the appropriate reference point of ‘fair representation’ should be (see e.g. Epstein 1996; Pot et al. 2021): representation of whom, of which experiences, and for what purposes? Moreover, increasing representational diversity in datasets or decision-making bodies does not automatically align genomic priorities with the actual needs, values, or lived realities of the people represented. Similarly, principles of fair distribution offer limited guidance in practice when benefits are diffuse, long-term, or collective, and when trade-offs between groups cannot be resolved without recourse to context-sensitive judgement rather than fixed rules.

In such settings, attempts to operationalise justice through formulaic models of justice, or direct reciprocity (‘tit for tat’) risk reproducing precisely the forms of exclusion and humiliation that solidarity seeks to avoid. What matters instead is the design of infrastructures that capable of listening, adapting, and responding over time, and institutions that treat participation not as compliance with predefined norms, but as an ongoing relational process grounded in respect for people’s dignity and agency. The more genomic systems invest in practices that actively prevent humiliation, accommodate plural needs, and avoid rigid interpretations of contribution and return, the more likely they are to produce outcomes that are experienced as equitable and legitimate by those affected.

This is also where care and solidarity perform distinct but complementary functions. Care is indispensable for recognising particular needs, vulnerabilities, and relationships; it keeps institutions attentive to the lived realities of those they serve. Yet, as argued above, care alone cannot sustain large-scale genomic systems, which require stable rules, durable infrastructures, and forms of cooperation that extend beyond face-to-face relationships. Solidarity provides this institutional architecture. By identifying – and acting on - the relevant commonalities that bind people despite their differences, it enables caring practices to be embedded in enduring arrangements, much as universal healthcare systems translate the recognition of shared vulnerability to illness into a collective commitment to care. In genomics, a solidarity-based infrastructure can similarly ensure that responsiveness to individual circumstances is not left to chance or personal goodwill, but becomes a reliable feature of the institutions through which genomic medicine is organised.

Such institutional design also matters not only for patients and their relevant others, but also for those working within genomic medicine. Research on moral injury shows that professionals experience profound ethical and psychological harm when institutional constraints prevent them from acting in accordance with their moral commitments, for example when they are required to enforce policies that undermine dignity, deny care, or instrumentalise people’s contributions (e.g. Čartolovni et al. 2021; Thibodeau et al. 2023; Wiinikka-Lydon 2019). Genomic systems that rely on narrow distributive logics or procedural compliance, rather than relational responsibility, increase the risk of moral injury not only among participants and communities but also among clinicians, counsellors, and researchers tasked with enacting these arrangements. By contrast, institutions oriented toward respect for everyone’s equal dignity and everyone’s capacity to contribute, that realise indirect reciprocity (defying transactional logics), and that avoid humiliation, create conditions under which both participants and professionals can recognise themselves as moral agents rather than functionaries within extractive or alienating systems. From this perspective, equitable genomics is less the result of getting representation or distribution right *ex ante*, than of building durable institutional capacities for attentiveness, reflexivity, and mutual recognition. Equity emerges not from abstract principles alone, but from infrastructures that are able to sustain dignity, trust, and shared responsibility in practice.

The infrastructure-dependence of genomic benefit becomes even more visible in Global South contexts. The H3Africa initiative was established to facilitate African-led genomics research, build local capacity, and address the underrepresentation of African populations in genomic datasets (de Vries et al. 2015; Sengupta et al. 2025). H3Africa has contributed substantially to research infrastructure, scientific leadership, and training. Moreover, many researchers and institutions involved in the initiative have made deliberate efforts to foster more solidaristic forms of genomics through equitable partnerships, local governance, capacity building, community engagement, and attention to benefit-sharing. At the same time, the initiative’s experience also illustrates the limits of what such efforts can achieve when access to basic healthcare remains uneven. In many participating countries, advanced genomic research infrastructures coexist with under-resourced health systems and fragile pathways from research to care. As a result, genomic discoveries may not translate into improved health outcomes for the populations whose data underpin the research. This does not mean that solidarity is absent from genomic research and medicine, but that its breadth and sustainability is conditioned by the broader institutional environment. Without robust healthcare infrastructures, the benefits generated through genomic research cannot be reliably converted into care, prevention, or lasting improvements in population health.

In this case, a solidarity-based response would not begin by asking how scarce resources should be allocated within health systems on the African continent alone. It would ask how institutions can be designed to recognise the contributions, shared interests, and mutual dependencies that link genomic research across regions. The first design principle of solidarity - the recognition of every person’s equal dignity and capacity to contribute - requires acknowledging that all researchers, research participants, patients, and communities are active contributors to its scientific advances, regardless of where they are based. They contribute data, expertise, labour, and the local infrastructures without which much contemporary genomic knowledge could not be produced. The second design principle, indirect reciprocity, suggests that these contributions need not be reciprocated immediately or by the same actors, but that they generate obligations within the broader global genomics ecosystem. Wealthier countries, institutions, and commercial actors that benefit disproportionately from contributions in less privileged world regions should therefore help strengthen the healthcare, research, and data infrastructures that enable genomic discoveries to translate into tangible health gains in the settings where the knowledge originated. Such support is not a matter of charity or development assistance but of sustaining a solidaristic arrangement in which benefits circulate across time and space. Finally, the principle of avoiding humiliation requires that such arrangements be organised as partnerships among equals rather than paternalistic transfers. Support should reinforce local capacities, leadership, and long-term stewardship, enabling all institutions and communities to shape the future of genomics on their own terms rather than remaining dependent on external actors.

## Beyond inclusion: applying solidarity and care to key domains of genomic practice

A solidarity-based lens draws attention to an aspect that is often overlooked in equity debates: people are already caring for and supporting one another through genomic practices. The central question, therefore, is not whether solidarity can be introduced into genomics research and practice, but how institutions can be designed to support these existing forms of mutual support rather than exploit, instrumentalise, or undermine them. As argued above, solidarity differs from justice in that it focuses less on the consistent application of abstract rules of fairness than on the collective organisation of practices through which people support one another and sustain the institutions on which they mutually depend. Solidarity complements care, in turn, in that it transforms responsiveness to others’ needs from a primarily dyadic relationship into a societal and institutional principle. It does so by grounding collective arrangements in shared vulnerabilities and mutual responsibility, rather than in one-directional obligations between care-givers and care-receivers.

Applied to genomics, solidarity entails building infrastructures that begin from the recognition that susceptibility to disease is a universal human condition, even if its manifestations are unevenly distributed. It implies organising the benefits and burdens of genomic knowledge according to principles of indirect reciprocity - where contributions circulate within the collective over time - rather than through transactional or market-based exchange. A solidaristic approach also requires genomic systems to be designed in ways that actively avoid humiliation – such as in the case of exclusion, paternalism, and often also charity - while sustaining the relational dimensions of genomic practices even as they are scaled institutionally. In this sense, solidarity provides a framework through which genomics can move beyond personalised risk management toward forms of collective health stewardship that are more just, inclusive, and sustainable.

A framework based on solidarity also challenges prevailing approaches to participation in genomics that focus primarily on recruitment and inclusion understood as numerical representation (e.g. Pot et al. 2021). Much of the existing literature and practice treats participation as the successful enrolment of individuals or groups into studies, biobanks, or datasets. While such efforts may improve diversity in genomic research, they often leave underlying power relations intact and risk instrumentalising participation as a means to access data rather than as a basis for shared decision-making.

Moreover, rather than centring participation on individual consent alone, solidarity foregrounds community-led agenda-setting and recognises that genomic priorities are shaped by social histories, structural inequalities, and collective experiences of harm or exclusion. This entails consent processes that are relational rather than transactional, acknowledging dependencies within families and communities as well as historical experiences of exploitation or neglect in biomedical research (e.g. Dove et al. 2017; Pratt et al. 2020; Shim et al. 2023). For participation to be genuinely meaningful, institutions must also invest in capacity building, including resources for community organisations, sustained engagement infrastructures, and opportunities to develop governance expertise. Crucially, solidarity requires recognising communities not merely as sources of data but as holders of situated knowledge and normative authority. Participation thus becomes less about accessing bodies or biospecimens and more about co-creating genomic futures through shared institutional arrangements.

### Data governance: from individual-level control to relational asset

And then there is data governance. Introducing solidarity into genomics also requires rethinking prevailing approaches that conceptualise genomic data as the (*de facto*, if not *de iure*) property or extension of individual persons. Such models, while offering important protections against misuse, are ill-equipped to address the relational nature of genomic data and their collective implications. Genomic data invariably exceed the individual: they reveal information about relatives, communities, and populations, and they gain value through aggregation, comparison, and linkage. A solidarity-based approach therefore treats genomic data as a relational asset whose governance must reflect shared vulnerabilities and collective interests.

In practice, this entails embedding governance rules that aim not merely to prevent harm but to advance public value (Prainsack et al. 2022). Such solidarity-based data governance seeks to ensure that benefits derived from genomic data accrue to the communities from which the data originate, rather than being captured predominantly by commercial actors (Carroll et al. 2020; Kukutai and Taylor 2016; Rainie et al. 2019; Walter et al. 2021). It requires transparent mechanisms of accountability and reciprocity, enabling affected groups to understand how data are used, to contest misuse, and to claim returns when collective value is generated. Federated and decentralised governance models are particularly well suited to this aim, as they allow data to remain under local or community control while still enabling collaboration and knowledge production across institutions. By contrast, extractive epistemologies - where data flow outward while benefits concentrate elsewhere - are directly challenged by a solidarity-based governance model.

### Benefit-sharing: indirect reciprocity and relational justice

The principle of indirect reciprocity offers a powerful conceptual and practical framework for rethinking benefit-sharing in genomics. Traditional benefit-sharing models often assume a direct, transactional relationship between contribution and return: individuals or communities provide data or samples and receive specific, identifiable benefits in exchange. In genomic research, however, such symmetry is frequently unrealistic. Benefits may materialise long after data collection, take non-monetary forms, or accrue at population level rather than the individual.

Indirect reciprocity reframes benefit-sharing as a relational commitment sustained over time rather than a discrete transaction. From this perspective, benefits need not mirror contributions in either form or timing. Instead, they may include investments in healthcare infrastructure, research capacity, education, or long-term access to genomic services. What matters is that contributions are recognised within a durable institutional framework that ensures communities are not left worse off and that gains generated through genomic research circulate back into the collective. By foregrounding long-term relationships and mutual responsibility, indirect reciprocity aligns benefit-sharing with relational justice rather than market exchange.

### Global genomics: solidarity as an antidote to postcolonial extraction

As noted, global genomic collaborations remain marked by profound asymmetries of power, resources, and recognition. In many cases, samples and data continue to flow from the Global South to the Global North, while scientific credit, infrastructural investment, and economic value flow in the opposite direction. While justice-oriented approaches may address some immediate ethical concerns, they are insufficient to confront these entrenched structural inequalities. A solidarity-based approach helps to address postcolonial dynamics in global genomics at the institutional level.

Solidarity begins from the recognition of our shared human vulnerability to disease, within communities as well as globally across communities, while acknowledging that exposure and capacity to respond are unevenly distributed. It calls for institutional architectures that facilitate reciprocity across borders, including co-governance arrangements, shared ownership of research agendas, and the equitable distribution of benefits. A solidaristic approach rejects humiliating or conditional forms of inclusion that frame Global South participation in terms of deficits or lack of capacity. Instead, it emphasises collective capability and mutual contribution, supporting forms of collaboration in which all parties participate as epistemic and moral equals. In this way, solidarity provides a principled and practicable basis for re-imagining global genomics in more just, reciprocal, and sustainable terms.

A solidarity-based framework that complements justice and care-based approaches reframes genomic equity not as a problem of distributing resources or access fairly, but as a question of institutional design. Rather than asking only how genomic benefits can be allocated fairly, it foregrounds how genomic systems are organised, who is recognised as a legitimate contributor, whose vulnerabilities count, and how responsibilities are shared over time. This shift has important ethical, methodological, and policy implications. It also highlights the particular relevance of community genetics as a field in which these implications can be realised in practice.

Such a solidarity-based framework moves beyond a focus on individual autonomy toward a conception of relational autonomy, understood as autonomy that is exercised within social relationships, dependencies, and institutional contexts. Equity, in this view, is not reducible to the fair distribution of genomic services or information but must be understood as structural: it concerns the background conditions that shape who can participate, who benefits, and who bears risks. Placing respect for everybody’s equal dignity, indirect reciprocity, and non-humiliation at the centre of genomic ethics shifts attention away from abstract choice and toward everyday institutional practices that can enable or undermine people’s standing as equal members of the societies that they are part of. Solidarity thus emerges not as an optional moral virtue, but as an institutional ethic that guides how genomic systems are designed, governed, and held accountable.

In addition, as noted, adopting a solidarity-based framework challenges approaches that treat participation as episodic recruitment or data extraction. It requires participatory design and co-production processes that engage people and communities as partners rather than sources of data, and it values qualitative, interpretive, and relational methods for understanding how genomics is experienced and enacted in everyday life. Longitudinal engagement becomes central, as trust, reciprocity, and mutual understanding cannot be built through once-off encounters. Studying genomics through this lens also entails attending to histories of marginalisation, power asymmetries, and prior encounters with institutions that shape how genomic initiatives are received and interpreted over time.

In policy terms, such an approach implies that genomic governance models should explicitly reflect shared vulnerability and collective responsibility, rather than individualised notions of risk, responsibility, and benefit. Consent processes must be contextual, relational, and ongoing, recognising that people’s circumstances, understandings, and concerns evolve. Benefits derived from genomics should therefore not be limited to individual returns, such as results or therapies, but should also accumulate at community and infrastructural levels, for example through strengthened healthcare services, local research capacity, or community-defined priorities. At the strategic level, national genomic policies should prioritise equity through solidarity, resisting competitive models that frame genomics primarily as an engine of national or commercial advantage.

Community genetics is particularly well positioned to operationalise this framework. As a field grounded in local contexts and shaped by sustained engagement with families and communities, it is inherently relational and attentive to social embedding. Community genetics regularly navigates the intersection of genetics, everyday life, care practices, and collective responsibility, and it already confronts the ethical complexities that arise at this intersection. Precisely because of its proximity to lived experience and its longstanding engagement with care, community genetics can become a leading domain for developing and demonstrating solidaristic genomic practices that are ethically robust, methodologically sound, and socially just.

## Conclusion

This paper has argued that prevailing approaches to equity in genomics, while necessary, are insufficient to address the relational, structural, and institutional conditions through which genomic practices are produced and experienced. Efforts to improve representation, widen access, or refine benefit-sharing mechanisms remain constrained as long as equity is conceptualised primarily as a justice-focused question concerned primarily with fair access and distribution. The latter risk leaving intact the political–economic arrangements, governance models, and epistemic hierarchies that continue to shape whose knowledge counts, whose vulnerabilities matter, and who ultimately benefits from genomic innovation.

By bringing care and solidarity into conversation, this paper offers an alternative ethical framework for rethinking justice in genomics. Care draws attention to the needs of specific individuals and groups, and to the lived realities of genomic participation: the relational labour, dependencies, and long-term responsibilities that genomic knowledge entails for individuals, families, and communities. At the same time, care alone cannot adequately guide the design of large-scale genomic institutions without risking paternalism or the loss of contextual sensitivity. Solidarity complements care by enabling these relational insights to be translated into durable institutional arrangements grounded in shared vulnerability, indirect reciprocity, and the prevention of humiliation. Together, care and solidarity shift the focus of equity in genomics from choice and allocation toward institutional responsibility, mutual accountability, and collective flourishing.

Applying this framework to participation, data governance, benefit-sharing, and global collaboration demonstrates that solidaristic genomic systems are neither abstract ideals nor distant aspirations. Elements of solidarity are already present in genomic practice, particularly where genomics is embedded in systems of universal healthcare and in the everyday work of community genetics. The challenge, therefore, is not to invent solidarity anew but to design institutions that sustain, scale, and protect these existing practices rather than undermine them through extractive, competitive, or individualising logics.

For community genetics in particular, this framework offers both affirmation and opportunity. As a field that is locally grounded, relational by design, and deeply engaged with the ethics of care, community genetics is uniquely positioned to lead the development of solidaristic genomic institutions. By foregrounding dignity, shared responsibility, and relational justice, it can help reorient genomics toward public value and democratic legitimacy.

Ultimately, the horizon of equity in genomics should not be limited to minimising harm or correcting imbalances after the fact. A solidarity-based approach invites a more ambitious aim: to build genomic infrastructures that actively contribute to just, caring, and resilient societies -ones in which genomic knowledge supports not only individual optimisation but collective well-being across time, place, and difference.

## Data Availability

No datasets were generated or analysed during the current study.

## Footnotes

1 I am grateful to Angus Clarke for helpful discussions on this point.
